# Supplementary material for: The interactome of intact mitochondria by cross-linking mass spectrometry provides evidence for coexisting respiratory supercomplexes
Source: Mol Cell Proteomics. 2017 Dec 8;17(2):216–32. doi: 10.1074/mcp.RA117.000470 (PMC5795388; doi:10.1074/mcp.RA117.000470)
Supplement: Supplemental Data [file supp_RA117.000470_133922_0_supp_23978_fzffwf.zip › spectra_annotation/mito_DR_spectra_annotation/260-1-3-1-5-1.pdf]

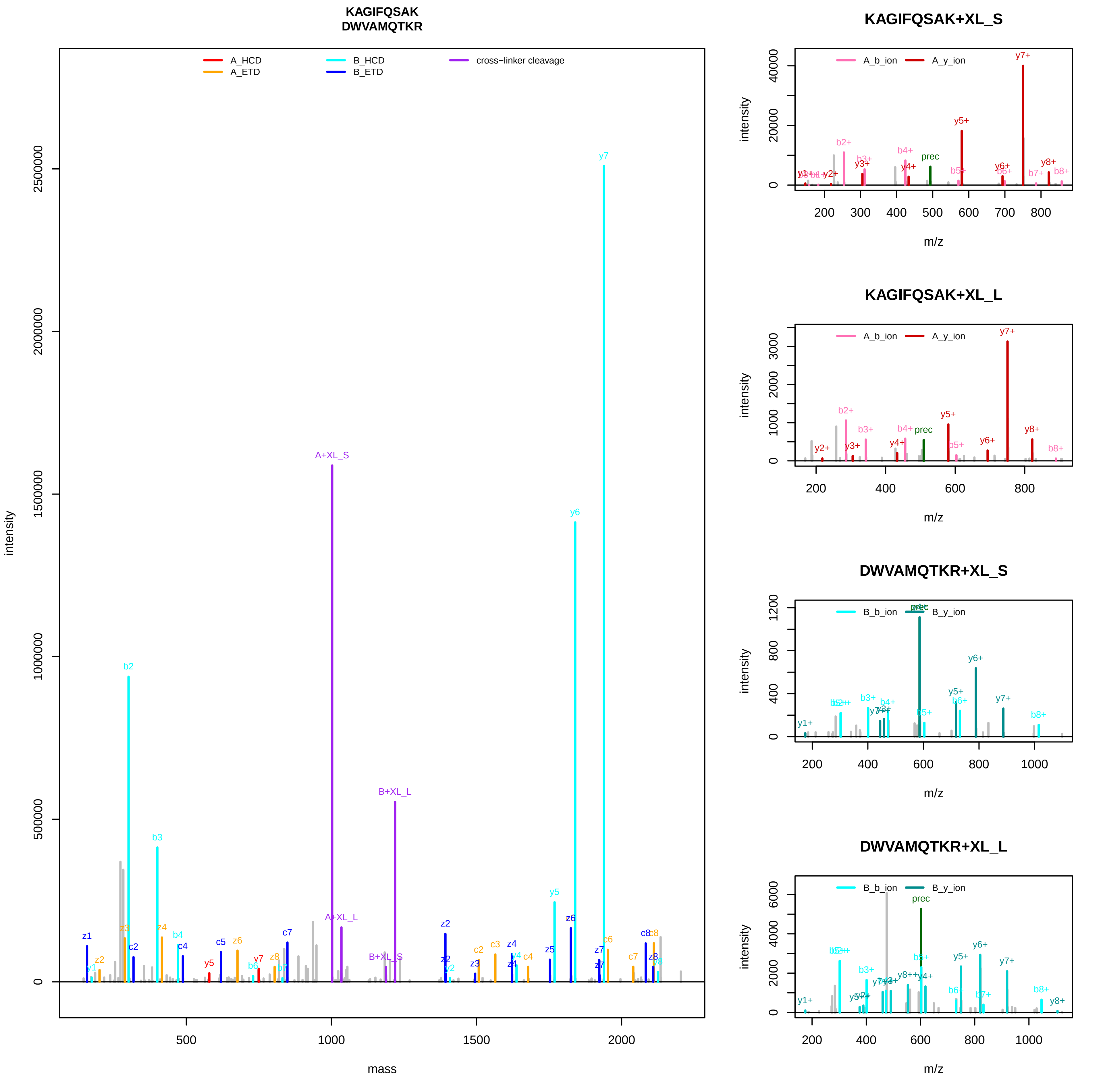
KAGIFQSAK
DWVAMQTKR
A_HCD
A_ETD
B_HCD
B_ETD
cross−linker cleavage
intensity
mass
0
500000
1000000
1500000
2000000
2500000
500
1000
1500
2000
y7
A+XL_S
y6
B+XL_L
b2
b3
y5
A+XL_L
B+XL_S
z6
c8
c7
c6
z8
z7
z5
z4
c4
c3
c2
z3
z2
y4
y2
c5
z6
y7
y5
z8
b6
b7
z4
b4
c4
c2
z3
z2
z1
y1
KAGIFQSAK+XL_S
KAGIFQSAK+XL_L
DWVAMQTKR+XL_S
DWVAMQTKR+XL_L
A_b_ion
A_y_ion
B_b_ion
B_y_ion
prec
m/z
0
20000
40000
1000
2000
3000
400
800
1200
4000
6000
200
300
400
500
600
700
800
1000
y7+
y5+
y6+
y8+
b8+
b7+
b6+
b5+
y4+
b4+
y3+
b3+
b2+
y2+
y1+
